# The impact of habitat fragmentation on tsetse abundance on the plateau of eastern Zambia^☆^

**DOI:** 10.1016/j.prevetmed.2009.05.009

**Published:** 2009-09-01

**Authors:** E. Ducheyne, C. Mweempwa, C. De Pus, H. Vernieuwe, R. De Deken, G. Hendrickx, P. Van den Bossche

**Affiliations:** a Avia-GIS, Risschotlei 33, 2980 Zoersel, Belgium; b Department of Veterinary and Livestock Development, Zambia; c Animal Health Department, Institute of Tropical Medicine, Antwerpen, Belgium; d Department of Veterinary Tropical Diseases, Faculty of Veterinary Science, South Africa

**Keywords:** Tsetse, GIS, Fragmentation analysis

## Abstract

Tsetse-transmitted human or livestock trypanosomiasis is one of the major constraints to rural development in sub-Saharan Africa. The epidemiology of the disease is determined largely by tsetse fly density. A major factor, contributing to tsetse population density is the availability of suitable habitat. In large parts of Africa, encroachment of people and their livestock resulted in a destruction and fragmentation of such suitable habitat. To determine the effect of habitat change on tsetse density a study was initiated in a tsetse-infested zone of eastern Zambia. The study area represents a gradient of habitat change, starting from a zone with high levels of habitat destruction and ending in an area where livestock and people are almost absent. To determine the distribution and density of the fly, tsetse surveys were conducted throughout the study area in the dry and in the rainy season. Landsat ETM+ imagery covering the study area were classified into four land cover classes (munga, miombo, agriculture and settlements) and two auxiliary spectral classes (clouds and shadow) using a Gaussian Maximum Likelihood Classifier. The classes were regrouped into natural vegetation and agricultural zone. The binary images were overlaid with hexagons to obtain the spatial spectrum of spatial pattern. Hexagonal coverage was selected because of its compact and regular form. To identify scale-specific spatial patterns and associated entomological phenomena, the size of the hexagonal coverage was varied (250 and 500 m). Per coverage, total class area, mean patch size, number of patches and patch size standard deviation were used as fragmentation indices. Based on the fragmentation index values, the study zone was classified using a Partitioning Around Mediods (PAM) method. The number of classes was determined using the Wilks’ lambda coefficient. To determine the impact of habitat fragmentation on tsetse abundance, the correlation between the fragmentation indices and the index of apparent density of the flies was determined and habitat changes most affecting tsetse abundance was identified. From this it followed that there is a clear relationship between habitat fragmentation and the abundance of tsetse flies. Heavily fragmented areas have lower numbers of tsetse flies, but when the fragmentation of natural vegetation decreases, the number of tsetse flies increases following a sigmoidal-like curve.

## 1 Introduction

Tsetse-transmitted trypanosomiasis is a major constraint to rural development in large parts of Africa (Swallow, 1998).

The tsetse flies occur in about 10 million km^2^ of sub-Saharan Africa and the trypanosomes they transmit can cause severe illness in livestock and people. The prevalence of bovine trypanosomiasis, transmitted by *Glossina morsitans morsitans* (Diptera: Glossinidae) the only species present, is about 30% (Simukoko et al., 2007). For their survival, tsetse flies are highly dependent on the presence of suitable habitat and hosts. The seasonal distribution of the flies is correlated with the distribution of its main host, cattle (Van den Bossche and Staak, 1997). However, in large parts of tsetse-infested sub-Saharan Africa the progressive clearing of the natural vegetation for cultivation, the introduction of domestic animals and the almost complete disappearance of large game animals have had important repercussions for the distribution and density of tsetse flies. In Malawi, for example, the distribution of tsetse flies is almost restricted to protected areas, where the vegetation is undisturbed whereas the extensive clearing of natural vegetation outside the protected areas has led to the disappearance of the tsetse flies and the disease they transmit (Van den Bossche et al., 2000). It can be anticipated that in the years to come and with continued population growth and the environmental change a similar decline in the distribution and density of the tsetse population and the disease prevalence will be observed elsewhere. This process of gradual reduction in challenge may in certain areas ultimately lead to autonomous, anthropogenic clearing of tsetse and thus the disappearance of the disease (Bourn et al., 2000; Reid et al., 2000). Understanding this process may contribute to the development of focused trypanosomiasis control strategies that exploit this autonomous tsetse clearing.

While the landscape structure has a strong potential to influence disease dynamics, the importance of landscape composition (number and type of patches) and configuration (spatial relationship among patches) is only beginning to be explored (Ostfeld et al., 2005). The quantification of the spatial heterogeneity of a landscape, also referred to as landscape fragmentation, is necessary to elucidate the relationship between the landscape and the ecological processes (Turner, 2005). A wide range of landscape fragmentation indices (or metrics) is now readily available to quantify the landscape composition and configuration for categorical data through dedicated software packages such as Fragstats (stand alone, http://www.umass.edu/landeco/research/fragstats/fragstats.html) or Patch Analyst (GIS-integrated, http://flash.lakeheadu.ca/∼rrempel/patch/).

Examples of landscape indices include the total area of a given land cover class, the number of patches of that land cover class, the shape of remaining land cover patches for composition, and the distance to the nearest patch of the same land cover class for landscape configuration. Often several indices are combined to completely quantify the entire landscape.

Even though the relationship between species diversity or species abundance and fragmentation characteristics is acknowledged, this relationship is not necessarily linear, and thresholds or minimum sizes for these factors may exist (Drinnan, 2005) below which species diversity or abundance declines more than linear. Determining these thresholds is important from a management/control perspective. To investigate the relationship between tsetse abundance and habitat fragmentation a study was initiated in the tsetse-infested livestock production area of the plateau of eastern Zambia where human population growth and agricultural expansion have brought about profound changes to the environment (Van den Bossche, 2001). Those changes have resulted in a substantial reduction in and fragmentation of the natural tsetse habitat and in the abundance of wildlife host.

## 2 Materials and methods

### 2.1 Study area

The study was carried out in an area situated between 31°47′ and 31°55′E (31.788–31.916E) and between 13°55′ and 14°12′S (13.916–14.12S) in Katete District, Eastern Province, Zambia. It is a highly cultivated area with a cattle population of approximately 8–10 animals/km^2^.

The vegetation in the study area can be classified into two main types.

**Fig. 1:**
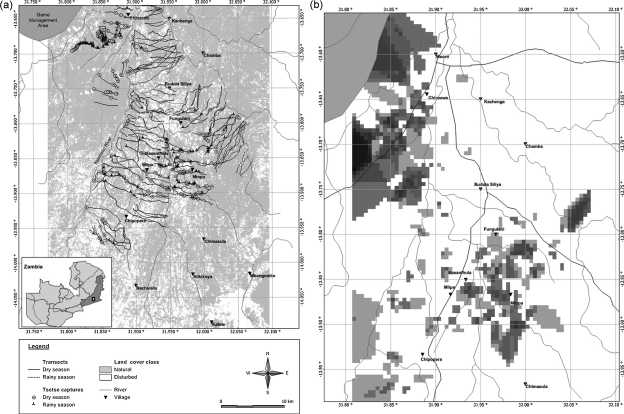
(A) Map of study area, location of fly-round transects and tsetse fly captures during the dry and wet season survey. (B) Map of index of apparent density, interpolated using kriging.

(i) Miombo, a one-storied open woodland with dominant genera *Brachystegi*a and *Julbernardia*, and which is found mainly on poorer soils on ridges or slopes and (ii) munga, a one- or two-storied woodland where the principal tree genera are *Acacia*, *Terminalia* and *Combretum*, which is associated with flat topography and which follows the streams and their smaller tributaries. Most villages are found in the miombo vegetation type. Tsetse flies are found in both vegetation types but their abundance in each of them varies seasonally (Van den Bossche and De Deken, 2002). The annual climatic cycle in the study area comprises three seasons: the warm rainy season (from early November to late April), the cold dry season (from early May to late August) and the hot dry season (from early September to late October). The study area is a subsistence farming area with maize and cotton being the most important crops. It represents a gradient of habitat change, starting from a zone with high levels of vegetation destruction and fragmentation as a result of human settlements and clearing for agriculture in the south and ending in an area where livestock and people are almost absent and where natural vegetation is largely untouched in the north (Fig. 1).

### 2.2 Tsetse distribution

To obtain up to date information on the distribution and density of *G. m. morsitans*, two surveys were carried out. The first survey was conducted during the rainy season (February/March 2006) and the second at the end of the hot dry season (October/November 2006). Use was made of the fly-round method along transects as described by Potts (1930) and Ford et al. (1959). According to this method a sample team of two men traversed a transect using a black screen (1.5 m × 1 m) baited with acetone released at approximately 200 mg/h (Shereni, 1984). The screen hung from a bamboo pole and was kept hanging vertically by weighting with a second bamboo pole at the bottom.

Each transect was about 6 km long and had about 30 sectors of roughly 200 m each. At the end of each sector was a stop. The fly-round team remained at each stop for 2 min and, using hand nets, captured tsetse alighting on the screen. All flies were killed immediately after capture. The fly-rounds started between 07:00 and 08:00 h or 15:00 and 16:00 h in the rainy and hot dry seasons, respectively. Records were kept of the number and sex of the tsetse captured at each stop along each transect.

All fly-round stops were georeferenced with a GPS using latitude–longitude coordinates (datum = WGS84). The dry season survey aimed at covering the same area as the one carried out during the rainy season. The starting points of each transect were identical but the position of the remainder of each transect could deviate slightly from its position during the rainy season survey. Moreover, during the dry season the area surveyed was extended to a zone that was not covered during the rainy season.

### 2.3 Land cover classification

Because of cloud contamination, only the dry season was available for selecting high quality satellite data. Due to the Scan Line Failure of the ETM+ sensor in May 2003, the most recent Landsat data was from 6 October 2002. Two consecutive Landsat scenes (Path/Rows = 170/069 and 170/070, respectively) were ordered through the GLOVIS portal from the United States Geological Survey (http://glovis.usgs.gov).

The two images were preprocessed separately prior to mosaicking to reduce any effects of differences in illumination and atmospheric absorption. The digital numbers were converted into near-surface reflectance taking into account atmospheric correction using a dark pixel subtraction method (Chavez, 1988). Subsequently, they were co-registered to remove any geometric distortions using a second order polynomial followed by a nearest neighbour resampling method. Finally, the imagery was mosaicked using a grey level matching method (Richards and Jia, 1999).

During the tsetse surveys, four different land cover types were identified, i.e. munga, miombo, agriculture and villages. In total, 560 reference points were georeferenced, this number was determined using the rule of thumb by Jensen (1996) who states the number of training sites should be >10*n*, distributed equally across the entire image. In the case, we need at least 10 × 7 = 70 training sites. For each point the landscape was described and pictures were taken. Using those ground truth data, training sites were delineated on the satellite imagery as input for a supervised classification. From these training sites, spectral signatures are derived for four land cover types and two additional classes (clouds, shadow). The satellite image was classified using a Gaussian Maximum Likelihood Classifier with equal prior probabilities for each spectral class. The accuracy assessment was performed using an independent validation set and was evaluated using a confusion matrix, the overall accuracy and the Kappa Index of Agreement, which represents the agreement between ground truth and prediction after removing the proportion of agreement that could be expected to occur by chance (Congalton, 2001). A Kappa Index of Agreement close to 1 indicates a high accuracy. All image processing was carried out in Idrisi Andes (www.clarklabs.org).

### 2.4 Fragmentation analysis

The classified image was reduced to two classes: “natural vegetation” comprising the miombo and munga land cover types and “disturbed vegetation” comprising the agriculture and villages land cover types. The classified image was overlaid with a grid of regular hexagons (De Clercq et al., 2007). Hexagons are selected because of their compact and regular form. Since habitat fragmentation occurs at different scales and to allow for the tsetse fly's mobility, two hexagon side sizes taking into account daily displacement capacity of *G. m. morsitans* (Hargrove, 2003) were used, i.e. 250 and 500 m. The landscape fragmentation was assessed using Patch Analyst in ArcView3.2 (www.esri.com) by calculating the following indices for each hexagon for the disturbed vegetation class: total class area, number of patches, mean patch size and patch size standard deviation. Based on these indices, the hexagons with similar fragmentation characteristics were grouped in classes using an unsupervised clustering method Partioning Around Mediods (PAM). In this method each point is initially randomly assigned to one of the clusters. During the subsequent iterative phase, the cost of the configuration is determined and the mediod is adjusted to the configuration that reduces the cost maximally. In essence, this is a hill-climbing search method. The iterative process finishes when no configuration with lower energy can be found. The cost is calculated as the distance from each point to the nearest mediod. The number of clusters in the dataset was determined by means of the Wilk's Lambda and the silhouette width in R Stats. Using the fragmentation indices for the mediod hexagon of each cluster, the clusters could be arranged according to fragmentation degree.

To link the entomological data to the fragmentation classes the index of apparent density (IAD) of tsetse was calculated for each polygon as follows:

$$IAD_{p}=\frac{NbCatches_{p}}{NbStops_{p}}$$

where *IAD*_*p*_ = index of apparent density for polygon *p*, *NbCatches*_*p*_ = number of flies captured in polygon *p*, *NbStops*_*p*_ = number of stops in polygon *p*.

Subsequently the IAD for all fragmentation classes was derived as the mean of the IAD of each polygon belonging to the fragmentation class. In order to test whether there is a significant difference between the different classes, the odds ratio of presence (i.e. IAD > 0) over absence (i.e. IAD = 0) was determined. In a next step, a general linear model with binomial distribution in R Stats was used to determine the difference between the categories.

## 3 Results

### 3.1 Tsetse distribution

The entire study area was covered using 238 transects (119 in the dry and 119 in the rainy season, respectively) consisting of 6645 stops. A total of 210 *G. m. morsitans* (140 male and 70 female flies) were captured during the dry season survey. During the rainy season survey, 182 flies (122 male and 60 female flies) were captured. The flies were captured at 304 stops located throughout the study area (Fig. 1).

### 3.2 Vegetation classification

Using the four vegetation types, the overall accuracy as tested with an independent validation set using the Gaussian Maximum Likelihood Classifier was 96%, while the Kappa Index of Agreement was 0.93. For all different vegetation types, except for miombo, a conditional Kappa Index of Agreement of more than 0.90 was obtained (agriculture = 0.9483, miombo = 0.7693, munga = 0.9580, villages = 0.9794, clouds = 1.0000, shadow = 0.9625). The lower conditional Kappa Index of Agreement for miombo was due to the misclassification of 17% of the miombo pixels as munga (Table 1). Since prior to the fragmentation analysis these two vegetation types were combined into a single ‘natural vegetation class’, the confusion between these two classes therefore did not affect the fragmentation analysis.

### 3.3 Fragmentation trends at different scales

In both cases the best clustering (lowest Wilk's lambda) was obtained for five clusters. All fragmentation indices contributed to the identification of the different fragmentation classes. However, it was noted that a subset of two indices (number of patches and class area) was sufficient to discriminate the clusters both at the 250 and 500 m scale. Fig. 3 illustrates the mediods per fragmentation class for both the 250 and 500 m scale.

At the 250 m scale (Fig. 3, top), there is a strong north–south axis of highly fragmented habitat along the road between Chipopera and Budula Siliya. Fragmentation decreases further away from this road in both directions. To the west, fragmentation becomes minimal when approaching the Nyamadzi river, and to the east close to protected forest west of Fungulani. Fragmentation is also more pronounced in the southern than in the northern part of the study area, especially in the area around Chipopera. Northwest of the Nyamadzi river, close to the game management area (GMA), the habitat is still intact.

**Fig. 2:**
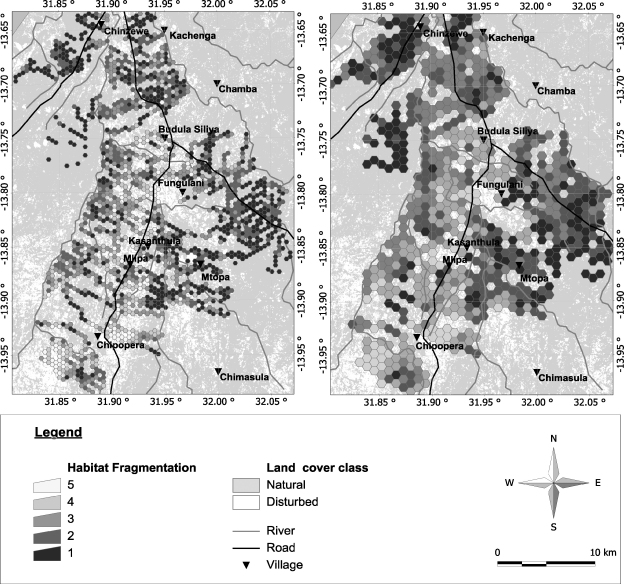
Representation of the degree of fragmentation of natural habitat in each of the fragmentation classes for hexagon side sizes of 250 m (left) or 500 m (right).

**Fig. 3:**
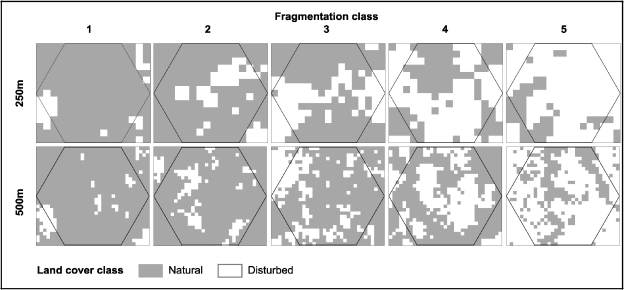
Mediods for each of the fragmentation classes for hexagon side sizes of 250 m (top) or 500 m (bottom).

**Fig. 4:**
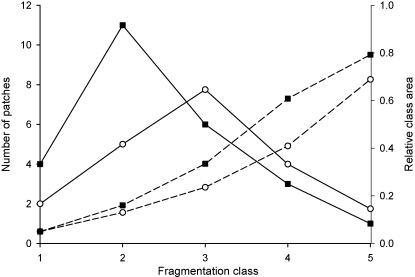
Number of patches (—) and class area (– – –) of disturbed vegetation in each of the habitat fragmentation classes for hexagon side sizes of 250 m (■) or 500 m (○). To allow for comparison between the two scales the number of patches at 500 m was divided by 4.

The same phenomenon can be observed at the 500 m (Fig. 3) scale but the fragmentation patterns observed at this scale seem more clustered. From Fig. 2 both the north/south (game management area vs. Chipopera/Katete) as well as the east–west gradient away from the road between Chipopera and Budula Siliya are observed. The protected forest north of Msoro appears as a non-fragmented zone as is the miombo/munga complex south of the GMA. Though the number of fragmentation classes is the same for both scales, the interpretation of the classes based on the mediods is slightly different (Fig. 4). While at the 250 m scale the hexagons with the highest degree of fragmentation (class 5) contain a single large patch of agriculture, hexagons with several (but a small number) of large patches are also included in this category at the 500 m scale. On the other hand, the hexagons with a fragmentation class 4 degree of fragmentation at the 250 m scale are similar to those which were assigned to fragmentation class 3 at the 500 m scale. Both categories show a considerable area of disturbed landscape consisting of large number of small patches. Finally at the 500 m scale, the clusters differentiated within the ‘lightly fragmented’ fragmentation class whilst these two categories are collapsed into one category at the 250 m.

### 3.4 Influence of fragmentation on tsetse abundance

**Fig. 5:**
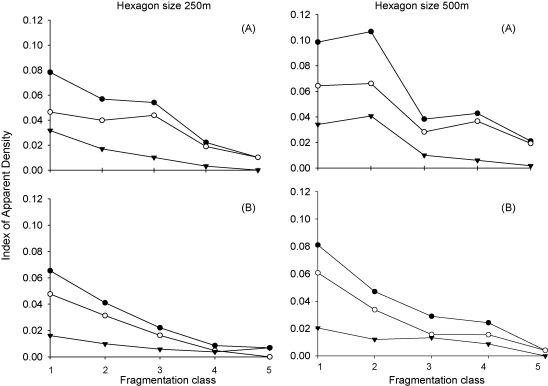
Index of apparent density of male and female tsetse (●), index of apparent density of males (○) and index of apparent density of females (▾) for each of the fragmentation classes and for hexagon side sizes of 250 and 500 m in dry season (A and B) and wet season (C and D).

Independent of the scale (250 and 500 m) the impact of fragmentation on tsetse abundance was similar (Fig. 5). During the dry as well as the rainy season the majority of the tsetse flies were captured in a non-fragmented landscape. Increasing levels of fragmentation resulted in a gradual decline in the IAD of male and female *G. m. morsitans* until a level of fragmentation (fragmentation class 4) is reached where the IAD is constant but low.

From Table 2 follows that based on the odds ratio fragmentation classes 1 (*α* = 0.99), 2 (*α* = 0.95) and 3 (*α* = 0.9) are significantly higher from the reference category (fragmentation class 5) (Table 3).

## 4 Discussion

### 4.1 Fragmentation quantification at different scales

The landscape fragmentation in the study zone can be characterized using two landscape indices that are easy to measure, i.e. class area and number of patches or by any other subset of landscape indices. This is due to the correlation between the estimated parameters.

At both scales the fragmentation trends are similar but they appear more clustered at 500 m due to the generalization of the landscape pattern within the hexagons. At the 250 m scale the trends are less obvious but the hexagons have a more homogeneous landscape within. This is simply caused by the smaller surface of the smaller hexagons.

### 4.2 Entomological repercussions

The outcome of this study shows that in the study area the destruction and fragmentation of the natural habitat of tsetse flies has significant repercussions for the density of those flies. Extensive clearing, mainly for cotton production in the southern part of the study area has resulted in the disappearance of large parts of the tsetse habitat and in a significant reduction in the apparent density of tsetse compared to the areas closer to the Luangwa escarpment where human density is much lower and the natural vegetation largely undisturbed. This reduction in the IAD of tsetse in the southern part can also be observed over time. Between 1990 and 1993, a longitudinal study monitoring the apparent density of the tsetse population in the southern part around Chipopera (Fig. 1), which is currently characterized by fragmentation classes 1 and 2, using identical sampling methods revealed an average monthly IAD of 0.64 flies during the rainy season (Van den Bossche and De Deken, 2002). This is substantially higher than the average IAD of 0.01 and 0.02 flies observed during the rainy season survey in the fragmentation classes 1 and 2 and at a scale of 250 m, respectively. Hence, over a period of about 15 years anthropogenic changes have resulted in the almost disappearance of tsetse from this area.

The results from this study suggest that the effect of habitat fragmentation on the apparent density of male and female tsetse flies is a gradual process with the IAD of tsetse increasing with decreasing levels of fragmentation. The results also show that this effect seems to be more pronounced in male flies. This difference between male and female flies is rather a result of a sampling bias inherent to the fly-round method that is less attractive to female compared to male *G. m. morsitans* (Vale and Phelps, 1978). The observed variations in the IAD occur between habitats with high levels of fragmentation or areas where tsetse are most likely to disappear autonomously and habitats with low levels of fragmentation or areas where the tsetse population is thriving and where control interventions are required to reduce its density.

Although suitable tsetse habitat is still present in all fragmentation classes it is expected that its degree of fragmentation affects the movements of tsetse flies in their quest for food. Since most of the wild hosts have disappeared from most of the study area, tsetse flies have become highly dependent on cattle for their survival (Van den Bossche and Staak, 1997). The cattle availability is highest in the highly fragmented landscape but distribution of cattle varies seasonally. Therefore tsetse are highly dependent on seasonal changes in their distribution to find their host (Van den Bossche and De Deken, 2002). All factors (especially habitat fragmentation) hindering those seasonal movements of the flies will thus have direct repercussions on the flies’ feeding success and their survival. The effect of those changes in apparent fly density on disease transmission requires further investigation.

### 4.3 Conclusions

The approach presented in this paper may form the basis for rationalizing area-wide tsetse fly control activities by identifying areas where tsetse flies are likely to disappear as a result of autonomous clearing and where control may not be required and identifying areas where tsetse flies are likely to persist and where control is thus more appropriate.

## Conflict of interest statement

None of the authors (Els Ducheyne, Cornelius Mweempwa, Claudia De Pus, Hilde Vernieuwe, Redginald De Deken, Guy Hendrickx, Peter Van den Bossche) has a financial or personal relationship with other people or organisations that could inappropriately influence or bias the paper entitled “The impact of habitat fragmentation on tsetse abundance on the plateau of eastern Zambia”.

## Figures and Tables

**Table 1:** Error matrix of classification: true land cover (columns) versus predicted land covers (rows). ErrorO: error of omission, the number of pixels that should have been added to the class but where omitted; ErrorC: error of commission, the number of pixels that were committed in the class but should have been added to another class.

	Truth							
	Agriculture	Miombo	Munga	Villages	Clouds	Shadow	Total	ErrorC
Predicted								
Agriculture	637	22	0	4	0	1	664	**0.041**
Miombo	26	646	65	0	0	3	740	**0.127**
Munga	0	140	3346	1	0	20	3507	**0.046**
Villages	5	1	0	248	0	0	254	**0.024**
Clouds	0	3	1	0	606	1	611	**0.008**
Shadow	0	0	0	0	0	725	725	**0.000**
Total	668	812	3412	253	606	750	6501	

ErrorO	**0.046**	**0.204**	**0.019**	**0.020**	**0.000**	**0.033**		**0.045**

**Table 2:** Odds ratio of presence over absence for the different fragmentation classes.

Fragm. class	Absence	Presence	Odds ratio
1	1962	137	0.07
2	1397	75	0.05
3	1466	52	0.04
4	698	12	0.02
5	240	3	0.01

**Table 3:** Statistical analysis of odds ratio using a general linear model with a binomial distribution.

	Estimate	S.D.	Error	*z*-Value	Pr(>|*z*|)
Frag 4	0.3187	0.6492	0.491	0.62349	
Frag 3	1.043	0.5972	1.746	0.08075	•
Frag 2	1.4574	0.5923	2.461	0.01387	*
Frag 1	1.7203	0.587	2.931	0.00338	**

Fragmentation class 5 (the class which is most fragmented) is the baseline category to which the other classes are compared.

• *p* < 0.1.

* *p* < 0.05.

** *p* < 0.01.
